# Transformation of the tumour microenvironment by a CD40 agonist antibody correlates with improved responses to PD-L1 blockade in a mouse orthotopic pancreatic tumour model

**DOI:** 10.18632/oncotarget.7610

**Published:** 2016-02-23

**Authors:** Nadia M. Luheshi, Jane Coates-Ulrichsen, James Harper, Stefanie Mullins, Michal G. Sulikowski, Philip Martin, Lee Brown, Arthur Lewis, Gareth Davies, Michelle Morrow, Robert W. Wilkinson

**Affiliations:** ^1^ MedImmune Ltd., Cambridge CB21 6GH, UK; ^2^ MedImmune LLC., Gaithersburg, MD 20878, USA

**Keywords:** CD40, PD-L1, pancreatic cancer, microenvironment

## Abstract

Despite the availability of recently developed chemotherapy regimens, survival times for pancreatic cancer patients remain poor. These patients also respond poorly to immune checkpoint blockade therapies (anti-CTLA-4, anti-PD-L1, anti-PD-1), which suggests the presence of additional immunosuppressive mechanisms in the pancreatic tumour microenvironment (TME). CD40 agonist antibodies (αCD40) promote antigen presenting cell (APC) maturation and enhance macrophage tumouricidal activity, and may therefore alter the pancreatic TME to increase sensitivity to immune checkpoint blockade. Here, we test whether αCD40 transforms the TME in a mouse syngeneic orthotopic model of pancreatic cancer, to increase sensitivity to PD-L1 blockade. We found that whilst mice bearing orthotopic Pan02 tumours responded poorly to PD-L1 blockade, αCD40 improved overall survival. αCD40 transformed the TME, upregulating Th1 chemokines, increasing cytotoxic T cell infiltration and promoting formation of an immune cell-rich capsule separating the tumour from the normal pancreas. Furthermore, αCD40 drove systemic APC maturation, memory T cell expansion, and upregulated tumour and systemic PD-L1 expression. Combining αCD40 with PD-L1 blockade enhanced anti-tumour immunity and improved overall survival versus either monotherapy. These data provide further support for the potential of combining αCD40 with immune checkpoint blockade to promote anti-tumour immunity in pancreatic cancer.

## INTRODUCTION

Tumours employ a number of mechanisms to escape detection and elimination by the adaptive immune system [1]. Tumour cells may directly escape T cell surveillance by downregulating expression and presentation of potentially immunogenic tumour-associated antigens [2, 3]. In addition, immunosuppressive mediators produced by tumour cells, stroma and tumour infiltrating leukocytes can drive effector T cell inactivation and exclusion of effector T cells from the microenvironment [4].

The PD-1 (programmed cell death protein 1) and CTLA-4 (cytotoxic lymphocyte-associated antigen 4) immune checkpoint pathways can both contribute to tumour immune evasion. PD-L1 (programmed death ligand 1) expressed on tumour cells and infiltrating myeloid cells engages PD-1 on activated T cells, downregulating T cell effector functions [5]. CTLA-4 on activated T cells binds to co-stimulatory molecules on antigen presenting cells, inhibiting further T cell activation and expansion, and facilitating suppression by regulatory T cells (T_reg_) [6]. Antibody therapies blocking PD-1, PD-L1 and CTLA-4 function enhance anti-tumour immunity, leading to durable clinical responses for a subset of patients with melanoma, lung cancer and other tumour types [7]. However, patients with pancreatic cancer, an aggressive disease with only a 7.2% 5 year survival rate, are reported to respond poorly to checkpoint blockade therapies [8, 9].

Melanoma patients that respond to PD-1 blockade are reported to show baseline PD-L1 expression and CD8^+^ effector T cell infiltration in their tumours [10–12]. This has led to the suggestion that PD-L1 / PD-1 blockade may be most effective where an existing anti-tumour CD8^+^ effector T cell immune response is actively being restrained by PD-L1 expression [7]. The pancreatic tumour microenvironment (TME) is dominated by a dense desmoplastic stroma infiltrated with immunosuppressive myeloid-derived suppressor cells, macrophages, fibroblasts and T_reg_ [13]. In contrast to melanoma tumours, effector T cells are often excluded from pancreatic tumours, and those that reach the TME appear inactive [14, 15]. The lack of response of pancreatic cancer patients to checkpoint blockade therapies has thus been proposed to be due to the establishment of the pancreatic TME as an “immune privileged” site [16].

Various strategies have therefore been developed to transform the immunosuppressive pancreatic TME, and so enhance the response of pancreatic cancer patients to immune checkpoint blockade therapies. A cell-based cancer vaccine, GVAX, was able to induce the formation of tertiary lymphoid aggregates in pancreatic cancer patient tumours, and resulted in objective clinical responses in combination with anti-CTLA-4 [17, 18]. In pre-clinical mouse models of pancreatic cancer (e.g. KPC model, KRAS^LSL-G12D/+^ / Trp53^LSL-R172H/+^ / Pdx-1-Cre) macrophage depletion with CSF1R inhibitors, or blockade of fibroblast-derived CXCL12 activity with a CXCR4 inhibitor, enhanced T cell infiltration and anti-tumour activity of αPD-L1/αPD-1 and αCTLA4 [19, 20].

CD40 agonistic antibodies (αCD40) in combination with gemcitabine have been reported to show early signs of clinical activity in pancreatic cancer patients [21]. CD40 agonism promotes macrophage and dendritic cell maturation, licenses cross-presentation of antigens to cytotoxic T cells, and promotes direct macrophage tumouricidal activity [22–24]. In both spontaneous genetic and transplantable KPC pancreatic tumour models, αCD40 given alone or in combination with chemotherapy inhibited tumour growth [25, 26]. Furthermore, the combination of αCD40 plus chemotherapy increased sensitivity to PD-1 / CTLA4 blockade, driving T cell-dependent anti-tumour immunity in a subcutaneous transplantable KPC model, and improving overall survival in the spontaneous KPC model [27].

Here we build on the above results by investigating the mechanism by which αCD40 improves sensitivity to PD-L1 blockade in pancreatic tumours. Since s.c. Pan02 tumour-bearing mice respond well to immune checkpoint blockade [28, 29], we developed a syngeneic orthotopic Pan02 model that better reflected the situation for patients and responded poorly to PD-L1 or CTLA-4 blockade. In contrast, we found that αCD40 delayed tumour growth and extended overall survival of orthotopic Pan02 tumour bearing mice. αCD40 treatment induced marked changes in the tumour immune microenvironment, promoting the formation of an immune cell rich capsule separating the tumour from the normal pancreas, enhancing Th1 chemokine expression and cytotoxic T cell infiltration, and increasing PD-L1 expression. The combination of αCD40 and αPD-L1 enhanced anti-tumour immunity and significantly improved overall survival. These data add further weight to the hypothesis that αCD40 can transform the immunosuppressive pancreatic TME, improving sensitivity to immune checkpoint blockade therapy.

## RESULTS

### The orthotopic Pan02 model of pancreatic cancer is insensitive to immune checkpoint blockade therapy but sensitive to αCD40

**Figure 1:**
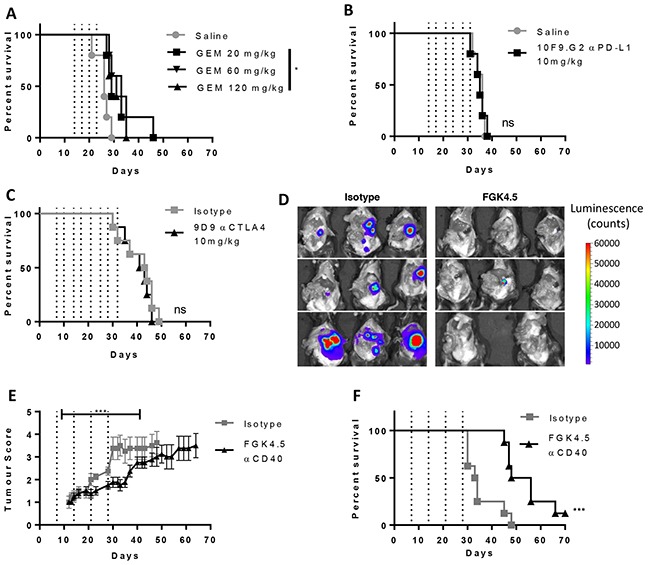
Orthotopic Pan02 model of pancreatic cancer is insensitive to immune checkpoint blockade therapy **A–C.** Effect of gemcitabine (A,GEM, days 14, 17, 20 and 23), αPD-L1 (B, 10F.9G2, 10 mg/kg, days 14, 17, 21, 24, 28 and 31) and αCTLA4 (C, 9D9, 10 mg/kg, days 7, 11, 14, 18, 21, 25, 28, 32) on overall survival in the orthotopic Pan02 model. n ≥ 5 mice per group. *p<0.05, ns not significant v.s isotype or saline control, log-rank Mantel-Cox test. **D.** Effect of CD40 agonist antibody FGK4.5 (i.p. 3 mg/kg, days 7, 14, 21, 28) on tumour burden at day 29 in Pan02-cag-luc2 tumour-bearing mice assessed in the exposed peritoneal cavity by bioluminescence imaging. **E.** Effect of FGK4.5 on tumour volume (blinded palpation scoring, see Supplementary Figure S2) of Pan02 tumour-bearing mice. Data are mean ± s.e.m. of n = 8 animals per group. *** p<0.001, statistical permutation test versus isotype control (see materials and methods for details). **F.** Overall survival of Pan02 tumour-bearing mice treated with 3 mg/kg FGK4.5 or isotype control. n = 8 animals per group ****p<0.0001 v.s isotype control, log-rank Mantel-Cox test.

A syngeneic orthotopic pancreatic tumour model was developed whereby Pan02 cells were implanted surgically into the pancreas tail of immunocompetent mice. Orthotopic Pan02 tumours diffusely invaded into the normal pancreas and metastasised to the peritoneal cavity. Primary tumours had a dense desmoplastic stroma highly infiltrated with macrophages (data not shown). We found that orthotopic Pan02 tumour-bearing mice responded poorly to gemcitabine, PD-L1 blockade and CTLA-4 blockade (Figure 1A–1C, study outlines in Supplementary Figure S1A). This allowed us to investigate whether αCD40 could increase sensitivity to PD-L1 blockade in this model.

In contrast to the minimal effects of immune checkpoint blockade, we found that a CD40 agonist antibody, FGK4.5, led to a significant reduction in early tumour growth and peritoneal metastatic spread (day 29) as measured by endpoint imaging of a luciferase-overexpressing Pan02 tumour cell line (Pan02-cag-luc2; Figure 1D, study outline in Supplementary Figure S1B). Using a standardised palpation scoring system to measure tumour growth (Supplementary Figure S2), we found that αCD40 significantly delayed tumour growth of both wild type Pan02 (Figure 1E) and Pan02-cag-luc2 tumours (Supplementary Figure S3) and delayed the onset of secondary indicators associated with disease progression (indicators described in Supplementary Figure S2A, data in Supplementary Figure S4). αCD40 also significantly extended overall survival (Figure 1F), in contrast to αPD-L1 and αCTLA4. However, many animals still ultimately succumbed to metastatic disease, suggesting that αCD40 alone was only partially successful in driving anti-tumour immunity.

### αCD40 transforms the TME

We next investigated the effects of αCD40 on the TME in Pan02 tumour-bearing mice. Pan02 tumour-bearing mice were treated with αCD40 (3mg/kg, once weekly from day 7 post-implantation). In separate studies (outlined in Supplementary Figure S1C), tumours were collected for immunohistochemistry at an early timepoint (day 22, 24h after third dose of αCD40), and at a later timepoint for mRNA and protein analysis (day 29, 24h after the last dose of αCD40).

**Figure 2:**
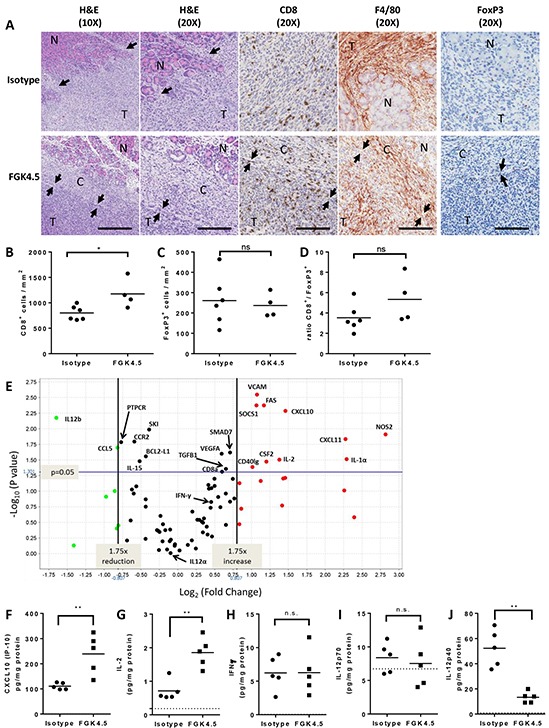
αCD40 transforms the TME **A.** Effect of FGK4.5 (3 mg/kg, day 7, 14, 21) on orthotopic Pan02 TME on day 22 was assessed by H&E staining, CD8, F4/80 and FoxP3 immunohistochemistry (IHC). Pan02 tumours (T) were highly invasive in isotype treated mice (top panels), and displayed multiple foci of invasion (arrows) into and destruction of the normal (N) recipient pancreas. In FGK4.5 treated mice (lower panels), tumours were encapsulated and separated from the normal pancreas (N) by a dense fibrous capsule (C) containing abundant inflammatory infiltrates. Arrows denote the clear border between the capsule (C) and the adjacent tumour (T). 10X scale bar = 100μm, 20X scale bar = 200μm. Images were captured from 10X Aperio™ scans or 20X scans (IHCs). **B–D.** Quantification of effect of FGK4.5 on CD8^+^ (B), FoxP3^+^ (C) and ratio of CD8^+^ / FoxP3^+^ T cells (D) in Pan02 tumours on day 22. n ≥ 4 animals per group, *p<0.05, ns not significant, Mann-Whitney test. **E.** Mean fold change in mRNA for immune-related genes in FGK4.5 treated Pan02 tumours (3 mg/kg day 7, 14, 21, 28) versus isotype treated tumours on day 29. mRNA expression quantified using a Taqman mouse immune array and normalised to six housekeeping genes. n ≥ 5 animals group. **F–J.** ELISA quantification of CXCL10 (F), IL-2 (G), IFNγ (H), IL-12p70 (I), and IL-12p40 (J) protein levels in Pan02 tumours on day 29 from mice treated with FGK4.5 (3 mg/kg, day 7, 14, 21, 28) or isotype control antibody. n ≥ 5 animals per group. **p<0.01, Mann-Whitney test.

We found that αCD40 caused a marked change in overall tumour morphology even at an early time-point (day 22). Isotype treated tumours were invasive, diffusely infiltrating the surrounding normal pancreas (Figure 2A). In contrast, αCD40 treated tumours were surrounded by a dense fibrous capsule with a marked inflammatory response, separating the tumour from the normal pancreas (Figure 2A). This inflammatory capsule contained both F4/80^+^ macrophages and CD8^+^ T cells (Figure 2A). Whilst there was some CD8^+^ cytotoxic T cell infiltrate in isotype treated tumours, this was also significantly enhanced in αCD40-treated animals (Figure 2B, 1.46 fold increase versus isotype control, p = 0.038). In contrast, αCD40 did not significantly change the number of tumour infiltrating FoxP3^+^ T_reg_ (Figure 2C). There was therefore a trend toward an improved ratio of CD8^+^ : FoxP3^+^ cells in αCD40-treated tumours (Figure 2D, 1.51 fold increase versus isotype, p=0.114).

Analysis of immune mediator expression in Pan02 tumours at day 29 revealed that αCD40 significantly upregulated the Th1 chemokine CXCL10 at the mRNA (Figure 2E) and protein (Figure 2F) level in Pan02 tumours, along with IL-2 (Figure 2E, 2G), perhaps explaining the expansion or increased infiltration of tumour CD8^+^ T cells. The Th1 mediators IFNγ (Figure 2E, 2H) and IL-12p70 (Figure 2I) were unchanged. Different effects were observed on the two subunits of IL-12p70 (IL-12p35 / IL12A and IL-12p40 / IL12B). Whilst IL-12p35 expression was unchanged (Figure 2E), expression of IL-12p40 was significantly downregulated by αCD40 at both the mRNA and protein level (Figure 2E, 2J). This may have led to a reduction in levels of IL-12p40 homodimers which can antagonise IL-12p70 effects [30]. αCD40-induced changes in tumour inflammatory mediators IL-1α, RANTES (CCL5) and GM-CSF (CSF2) at the mRNA level (Figure 2E) did not translate into significant increases in protein expression (data not shown). Thus, αCD40 significantly altered the TME, enhancing CD8^+^ T cell infiltration, promoting encapsulation of tumours by an immune infiltrate, and driving a partial Th1 shift in the cytokine / chemokine milieu.

### αCD40 drives myeloid cell maturation and memory T cell expansion in spleen

**Figure 3:**
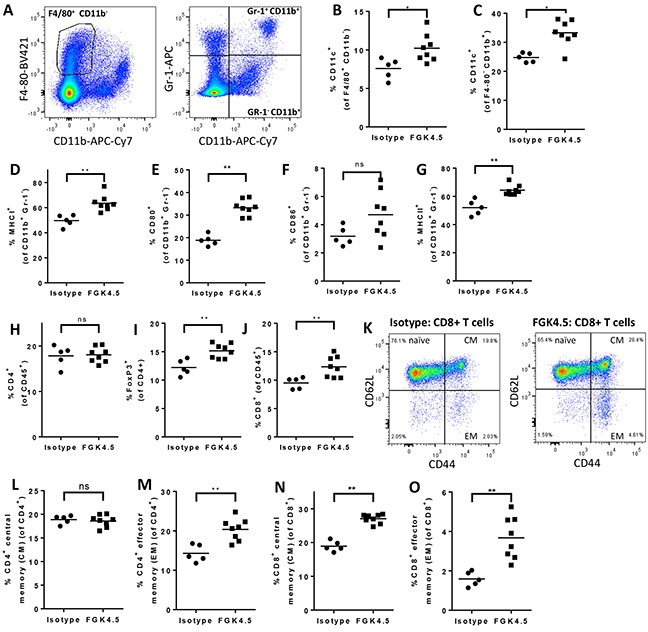
αCD40 drives myeloid cell maturation and memory T cell expansion in spleen **A.** Gating strategy for identification of F4/80^+^ CD11b^−^ (left panel), CD11b^+^ Gr-1^−^ and CD11b^+^ Gr-1^+^ (right panel) myeloid cells in splenocytes from Pan02 tumour-bearing mice. **B, C.** Effect of FGK4.5 treatment (i.p. 3 mg/kg day 7, 14, 21, 28, endpoint 24h after last dose) on CD11c expression in splenic F4/80^+^ CD11b^−^ (B) and F4/80^−^ CD11b^+^ (C) myeloid cells. **D–G.** Effect of FGK4.5 on splenic CD11b^+^ Gr-1^−^ myeloid cell expression of MHCI (D), CD80 (E), CD86 (F) and MHCII (G). **H–J.** Effect of FGK4.5 treatment on the proportions of total CD4^+^ (H), CD4^+^ FoxP3^+^ (I) and CD8^+^ (J) T cells in Pan02 tumour-bearing mouse spleens. **K.** Example data showinggating strategy fornaïve, CM and EM spenic CD8^+^ T cell populations in splenocytes from animals treated with isotype (left panel) or FGK4.5 (right panel). **L–O.** Effect of FGK4.5 treatment on the proportions of CD4^+^ (L, M) and CD8^+^ (N, O) cells with a central memory (CM, CD44^+^ CD62L^+^, L, N) or effector memory (EM, CD44^+^ CD62L^−^, M, O) phenotype. n ≥ 5 animals per group. **p<0.01, *p<0.05 Mann-Whitney test.

In order to investigate the effects of αCD40 on the systemic immune system, Pan02 tumour-bearing mice were treated with αCD40 (once weekly from day 7 post implantation, four doses). Mice were sacrificed 24h after the final dose of αCD40 (day 29, study outline Supplementary Figure S1C), and splenic myeloid and T cell populations were characterised by flow cytometry (Figure 3, and Supplementary Figure S5). Three principal groups of splenic myeloid cells were defined based on F4/80, CD11b and Gr-1 expression (Figure 3A). The CD11b^+^ / Gr-1^−^ gate captures multiple splenic myeloid cell populations including monocytes, macrophages and dendritic cells [31]. The CD11b^+^ Gr-1^+^ gate captures immunosuppressive myeloid-derived suppressor cells, as well as inflammatory monocytes and neutrophils [32]. The CD11b^−^ F4/80^+^ gate identifies splenic red pulp macrophages involved in senescent red blood cell clearance [33]. Only small changes were observed in the proportions of splenic myeloid cells from mice treated with αCD40 (Supplementary Figure S5A–S5C). However, αCD40 treatment induced a marked increase in the expression of markers of mature antigen presenting cells on these cell populations (Figure 3B–3G, Supplementary Figure S5D-S5K), suggesting a change in the differentiation status of splenic myeloid cells. The dendritic cell marker CD11c was upregulated on both F480^+^ and CD11b^+^ cell populations (Figure 3B, 3C). Induction of MHCI, MHCII, and of the co-stimulatory molecules CD80 and CD86 was also observed broadly on all myeloid populations (Figure 3D–3G, Supplementary Figure S5D–S5K). The only exception to this general trend was a slight downregulation of MHCI expression on CD11b^+^ Gr-1^+^ cells (Supplementary Figure S5H). In addition, αCD40 treated mice showed expansion of FoxP3^+^ T_reg_ and CD8^+^ cytotoxic T cells (Figure 3I, 3J), and of memory subsets of both CD4^+^ and CD8^+^ T cells (Figure 3K–3O). Thus, αCD40 drove maturation of splenic myeloid cells towards an antigen presenting cell phenotype, and expansion of T_reg_ and memory CD4^+^ and CD8^+^ T cells. These data are consistent with αCD40 driving systemic immune activation, in addition to transforming the TME.

### αCD40 upregulates PD-L1 in tumour and spleen

**Figure 4:**
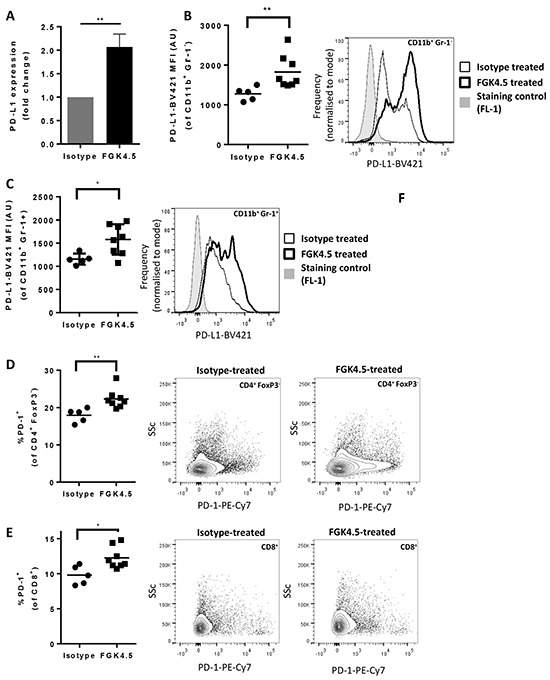
αCD40 upregulates PD-L1 in tumour and spleen **A.** Effect of FGK4.5 treatment (i.p. 3 mg/kg day 7, 14, 21, 28, endpoint day 29) on PD-L1 mRNA expression in Pan02 tumours. Data shown are fold change in expression versus isotype treated tumours, with expression levels normalised to the housekeeping gene Hprt. Data are mean ± s.e.m of n ≥ 5 animals per group **p<0.01 Mann-Whitney test. **B, C.** Effect of FGK4.5 treatment on PD-L1 expression on CD11b^+^ Gr-1^−^ (B) and CD11b^+^ Gr-1^+^ (C) myeloid cells in spleen. Scatter plots (left panel) show geometric mean fluorescence intensity of cells stained with PD-L1-BV-421 from individual mice. Histograms (right panel) show example PD-L1 staining in splenocytes from a representative animal treated with FGK4.5 (thick line) or isotype (thin line) in comparison to fluorescence minus 1 (FL-1) staining control (shaded histogram). n ≥ 5 animals per group **p<0.01, *p<0.05 Mann-Whitney test. **D–F.** Effect of FGK4.5 on PD-1 expression on CD4^+^ FoxP3^−^ (D) and CD8^+^ (E) T cells in spleen. Scatter plots (left panel) show % PD-1 positive cells from individual animals. Contour plots (centre and right panels) show PD-1 staining in splenocytes from a representative animal treated with isotype (centre panel) or FGK4.5 (right panel). n ≥ 5 animals per group **p<0.01, *p<0.05 Mann-Whitney test.

Since induction of PD-L1 expression was reported to mediate acquired resistance to αCD40 in breast tumour models [34], we next investigated whether αCD40 induced PD-L1 and PD-1 expression in the orthotopic Pan02 model. 24h after the last dose of αCD40 (day 29, study outline in Supplementary Figure S1C), tumour PD-L1 expression was quantified by qPCR, and splenocyte PD-L1 and PD-1 expression was quantified by flow cytometry. αCD40 upregulated PD-L1 mRNA expression in Pan02 tumours (Figure 4A, mean 2.1 fold increase in expression, p<0.01 versus isotype). In the spleen, αCD40 also upregulated PD-L1 on CD11b^+^ Gr-1^−^ and CD11b^+^ Gr-1^+^ myeloid cells (Figure 4B, 4C). Furthermore, PD-1 expression was induced by αCD40 on splenic CD4^+^ FoxP3^−^ (Figure 4D) and CD8^+^ (Figure 4E) effector T cells, but not on CD4^+^ FoxP3^+^ T_reg_ (data not shown).

### αCD40 + αPD-L1 combination improves anti-tumour immunity

**Figure 5:**
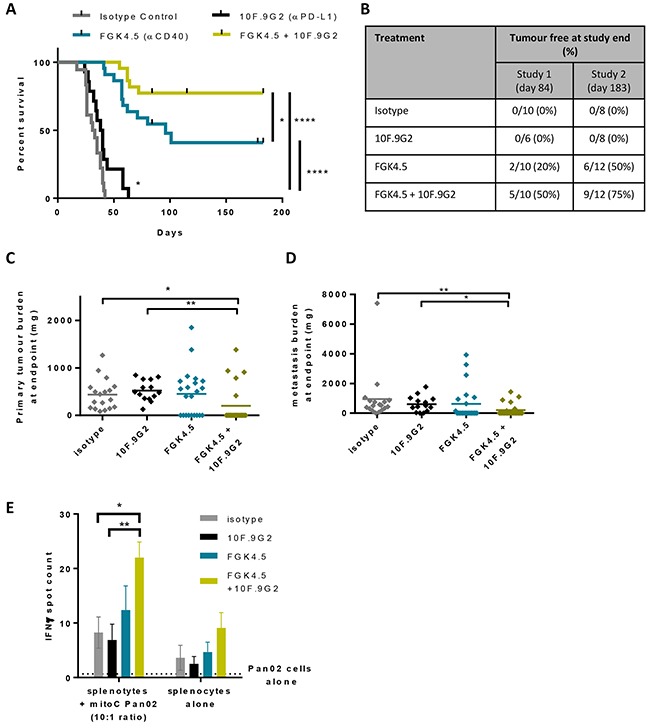
αCD40 + αPD-L1 combination improves anti-tumour immunity **A.** Effect of FGK4.5 (i.p. 3 mg/kg days 7, 14, 21, 28) and 10F.9G2 (i.p. 10 mg/kg days 7, 10, 14, 17, 21, 24) on the overall survival of mice bearing Pan02 tumours. Data are compiled from n = 2 independent experiments with a total of n ≥ 14 animals per group.****p,0.0001, *p<0.05, log-rank Mantel-Cox test. **B.** Proportion of mice that were tumour free at study end for n = 2 independent studies. **C, D.** Quantification of Pan02 primary tumour burden (C) and metastasis burden (D) at endpoint. n ≥ 14 animals per group from n = 2 independent experiments. **p,0.01, *p<0.05, Kruskal-Wallis with post-hoc Dunn's multiple comparison test. **E.** Quantification of the effects of FGK4.5 and 10F.9G2 on the number of IFNγ positive splenocytes following *in vitro* re-stimulation with Pan02 cells. Data are mean ± s.e.m. of n ≥ 4 animals per group, **p<0.01, *p<0.05, Two way ANOVA with post hoc Sidak's multiple comparisons test.

Because anti-CD40 treatment increased CD8^+^ T cell infiltration and also upregulation of PD-1 and PD-L1, we tested the hypothesis that a combination of αCD40 and αPD-L1 would induce systemic anti-tumour immunity in this model. Pan02 tumour-bearing mice were treated with αCD40 (3 mg/kg, once weekly from day 7, four doses) and αPD-L1 (10 mg/kg, twice weekly from day 7, six doses), and monitored for tumour growth and overall survival (study outline Supplementary Figure S1D). Primary tumour and peritoneal metastatic tumour burden was quantified for all animals at endpoint. Following on from an initial study running for 84 days, a second, extended study was performed to evaluate long term survival out to 6 months. When PD-L1 blockade was initiated at day 7 (Figure 5A) rather than day 14 (Figure 1C, compare study outlines in Supplementary Figure S1A and S1D), a minimal improvement in overall survival was observed (Figure 5A). The combination of αCD40 and αPD-L1 significantly increased overall survival in comparison to either treatment alone (Figure 5A, p<0.0001 versus isotype control or αPD-L1, p<0.05 versus FGK4.5). In the second, extended study 75% animals treated with this combination survived for 6 months after tumour implantation (Figure 5B). A larger proportion of the animals in the combination group were tumour free at endpoint by examination of the abdominal cavity after sacrifice (Figure 5B), and by quantification of dissected primary and metastatic tumour burden (Figure 5C, 5D). In order to investigate whether αCD40 / αPD-L1 treatment had induced a systemic anti-tumour immune response, animals treated with these agents individually or in combination were sacrificed 24h after the last dose of αCD40, and splenocytes were re-stimulated with Pan02 cells. An increased number of Pan02-reactive splenocytes were detected by IFNγ ELISPOT assay in animals treated with the combination of αCD40 and αPD-L1 (Figure 5E). Taken together, these data indicate that αCD40 combination with αPD-L1 enhanced systemic anti-tumour immunity in the orthotopic Pan02 model.

## DISCUSSION

We report here that a CD40 agonist antibody transformed the TME and promoted systemic anti-tumour immune activation in a mouse orthotopic pancreatic tumour model, Pan02. αCD40 treatment improved overall survival as a single agent, and increased sensitivity to PD-L1 blockade, leading to long term survival and enhanced anti-tumour immunity. Our data build on previous reports that αCD40 increased sensitivity to immune checkpoint blockade in mouse pancreatic (s.c. transplantable and spontaneous KPC) and breast tumour models [27, 34]. We demonstrate the effects of this combination in a pancreatic orthotopic tumour model and provide further insight into the mechanism by which αCD40 improves sensitivity to αPD-L1.

We found that orthotopic Pan02 tumour-bearing mice were resistant to PD-L1 and CTLA-4 blockade. However, CD8^+^ cytotoxic T cells were not excluded from isotype treated Pan02 tumours, suggesting that Pan02 tumour-infiltrating CD8^+^ T cells are either naïve or have become inactivated. Both exclusion of effector T cells from tumours and inactivation of tumour infiltrating T cells have been proposed to be responsible for the failure of some patients to respond to immune checkpoint blockade [4]. CD8^+^ T cells are excluded from many pancreatic cancer patient tumours, and from KPC mice with spontaneous pancreatic tumours [14, 15]. However, in pancreatic cancer patients where CD8^+^ T cell infiltration in tumours is observed, CD8^+^ T cells become inactivated through loss of CD3ζ [14].

Multiple effector mechanisms have previously been reported to contribute to αCD40-mediated inhibition of tumour growth and metastasis. αCD40 can induce both T cell-independent anti-tumour effects, enhancing macrophage tumouricidal activity and stromal remodelling, and CD8^+^ T cell-dependent anti-tumour immunity [35]. Our data are more consistent with αCD40 having induced both innate and adaptive immune activation in Pan02 tumour-bearing mice to improve overall survival. Treatment with a CD40 agonist antibody drove the formation of a dense capsule rich in cytotoxic T cells and macrophages that separated the pancreatic tumour from the normal pancreas. Furthermore, αCD40 upregulated IL-2 and the Th1 T cell chemokines, CXCL10 and CXCL11, and increased CD8^+^ T cell infiltration and tumour PD-L1 expression. Our finding that αCD40 induced an increase in CD8^+^ cells in orthotopic Pan02 tumours is in accord with data reported by Zippelius *et al*. [34] in s.c. MC38 breast tumours. αCD40 also drove systemic innate immune activation (APC maturation), and expanded central and effector memory cytotoxic T cells.

The majority of αCD40-treated animals developed progressive disease, indicating that αCD40-induced adaptive immune activation was insufficient. Our data suggest that upregulation of the PD-L1 / PD-1 immune checkpoint, both in the TME and systemically on myeloid cells, contributes to the failure of this adaptive immune response. Thus, whilst PD-L1 blockade alone had minimal effects on overall survival, combining CD40 agonism with PD-L1 blockade improved overall survival in comparison to either monotherapy alone.

PD-L1 upregulation on tumour cells (including Pan02 cells) and myeloid cells can be induced by IFNγ [6, 28]. However, we did not observe any increase in IFNγ in the TME with αCD40 treatment. It is possible that IFNγ induction occurred at an earlier time point and had since returned to baseline. Alternatively, PD-L1 may have been induced via an IFNγ-independent pathway in this model [36-38].

Survival times for pancreatic cancer patients treated with even the more recently developed chemotherapy regimens (FOLFIRINOX, or gemcitabine plus nab-paclitaxel) remain poor [39, 40]. Chemotherapy could theoretically promote anti-tumour immune responses by inducing immunogenic cell death and depleting suppressive immune cells [41-43]. However, it is also possible that the immunosuppressive effects of chemotherapy would limit responses to immunotherapy. In a spontaneous mouse pancreatic tumour model, the addition of gemcitabine did not improve the anti-tumour efficacy of CD40 agonism [25]. This contrasts with an earlier report of the benefits of this combination in less immunosuppressive subcutaneous tumour models [44]. The combination of nab-paclitaxel, gemcitabine and αCD40 with αPD-1 improved responses versus αPD-1 alone in the KPC model. However, the contribution of chemotherapy to this effect was not investigated [27]. We found that αCD40 increased sensitivity to PD-L1 blockade in the absence of chemotherapy in an orthotopic pancreatic tumour model. A similar combination effect in the absence of chemotherapy was also observed in breast tumour models [34]. Thus the potential benefits of adding chemotherapy to a combination with αCD40 and αPD-L1 treatment remain unresolved.

In summary, our data provide further preclinical support for the potential benefits of combining immunotherapies such as αCD40 that transform the TME with immune checkpoint blockade to promote anti-tumour immunity in pancreatic cancer.

## MATERIALS AND METHODS

### Cell lines and reagents

Pan02 cells, a SMAD4-null chemically induced mouse pancreatic tumour line, was from the National Cancer Institute [45, 46]. Pan02-CAG-luc2 cells were generated by lentiviral transduction. Briefly, DNA encoding the luciferase 2 gene (luc2, Promega) was cloned into a lentiviral vector (pCDH-CAG-IRES-puro, System Biosciences). Lentivirus was generated using the pPack lentiviral packaging system (SBI), and used to transduce Pan02 cells. Following antibiotic selection, a pool of stably transduced cells (Pan02-CAG-luc2) was obtained and used for in vivo studies. Luc2 expression in the Pan02-CAG-luc2 cell line pool was stable up to passage 16, as determined by luciferase assay (Promega, data not shown).

The following antibodies from eBioscience, BD Bioscience and Biolegend were used for flow cytometry: anti-CD45 (clone 30-F11), anti-CD44 (clone IM7), anti-CD62L (clone MEL-14), anti-CD3 (clone 145-2C11), anti-CD4 (Clone RM4.5), anti-CD8 (clone 53-6.7), anti-F4/80 (clone BM8), anti-MHCI (clone 28-8-6), anti-CD80 (clone 16-10A), anti-CD86 (clone GL-1), anti-MHCII (clone M5/114.15.2), anti-Gr-1 (clone RB6-8C5), anti-CD11b (clone M1/70), anti-CD11c (clone N418), anti-CD19 (clone6D5), anti-PD-L1 (clone 10F.9G2), anti-PD-1 (clone 29F.1A12), anti-FoxP3 (FJK-16S).

### Murine syngeneic orthotopic pancreatic Pan02 pancreatic tumour model

Experiments using 6 - 8 week old female C57BL/6J mice (Charles River) were conducted under a U.K. Home Office Project Licence in accordance with the U.K. Animal (Scientific Procedures) Act 1986 and in accordance with EU Directive EU 86/609. 30 minutes after prophylactic analgesia (buprenorphine, 60 μg s.c.), mice were shaved and anaesthetised (isoflurane). 5 × 10^5^ Pan02 or Pan02-CAG-luc2 pancreatic tumour cells in chilled matrigel (Corning) were surgically implanted in the pancreas tail [47] and the abdominal wall was closed with dissolvable sutures and autoclips. Clips were removed seven to 10 days after surgery.

Group sizes for tumour growth studies were determined based on existing data for orthotopic Pan02 tumour growth, to test for greater than 45 % tumour growth inhibition. The power calculation was a one-sided 2 sample t-test carried out at the 5 % significance level. Animals were randomised to treatment groups based on body weight prior to the first treatment. Due to the technical challenges of in vivo imaging of orthotopic tumours in C57/BL6 mice, we developed and validated a blinded scoring system based on abdominal palpation and animal welfare (Supplementary Figure S2). This incorporates both a score for primary tumour size by abdominal palpation (score of 1-6) and clinical observations (weight loss, tumour attachment to abdominal wall, secondary tumours, abdominal swelling and eschar formation, all adding +1 to overall score). For survival studies, animals were euthanized when either their primary tumour or combined tumour and clinical observation score reached +6. FGK4.5 CD40 agonistic antibody (rat IgG2a, 3 mg/kg, 4 doses, once weekly from day 7), 9D9 CTLA-4 blocking antibody (mouse IgG2b, 10 mg/kg, day 7, 11, 14, 18, 21, 25, 28, 32) and 10F.9G2 PD-L1 blocking antibody (rat IgG2b, 10 mg/kg, 6 doses, twice weekly from day 7 or day 14 as indicated) were from Bio X Cell. All antibodies were dosed intraperitoneally (i.p).

For imaging studies, luciferase expression in Pan02-CAG-luc2 tumours was assessed 29 days after cells were implanted. Animals were injected with 3.3 mg of luciferin (Perkin Elmer) i.p., euthanised and the peritoneal cavity exposed. The peritoneal cavity was imaged 20 minutes after luciferin injection on the IVIS100 (Perkin Elmer) with an acquisition time of 10 seconds.

### Flow cytometry analysis

Animals were euthanized and spleens were removed and dissociated to a single cell suspension through a 70 μm nylon mesh. Splenocytes were stained with a fixable live dead dye (Life Technologies) followed by block with anti CD16/32 antibody (eBiosciences) and staining with cocktails of fluorescence conjugated antibodies prior to fixation in 1 % formaldehyde / PBS. FoxP3 nuclear staining was carried out on fixed and permeabilised cells (FoxP3 / transcription factor staining buffer set, eBioscience). Stained cells were analysed by flow cytometry (BD LSRFortessa, BD Biosciences) with data analysis in FlowJo (Tree Star). Positive staining gates were identified by comparison to cells stained with the full antibody panel minus the antibody of interest (FL-1 control).

### Immunohistochemistry and image analysis

Tumour samples were harvested, fixed in 10 % neutral buffered formalin, processed and embedded into paraffin wax blocks. Sections were taken at 4 μm and then de-waxed, rehydrated and antigen retrieved using either a pressure cooker with low or high pH buffer (Vector / Dako) or enzymatic retrieval with proteinase K. Sections were then blocked for endogenous peroxidase with H_2_O_2_ in methanol prior to immunostaining. Following a protein/serum block, sections were immunostained with primary rat monoclonal antibodies to FoxP3 (eBioscience), CD8 (eBioscience) and F4/80 (AbD Serotec) for 1 hour at room temperature. Primary antibodies were detected with goat anti-rat IgG (Jackson ImmunoResearch) followed by anti-goat Ig ImmPRESS-HRP polymer (Vector). CD8 was detected directly with mouse adsorbed anti-rat ImmPRESS-HRP polymer (Vector). Staining was then visualised using DAB+ substrate (Dako). Sections were then counterstained with Haematoxylin, dehydrated, cleared and permanently coverslipped. Tissue morphology and collagen were also demonstrated using Haematoxylin & Eosin and Masson's Trichrome staining respectively. Slides were digitally scanned using an Aperio Scanscope and whole slide image analysis was performed in the tumour region only with algorithms created in Definiens Tissue Studio version 4.1.

### Tumour mRNA and protein analysis

Animals were euthanized and pancreatic tumours dissected out from normal pancreas, before being either snap frozen or transferred to RNALater. Snap frozen tumours were homogenised in tissue lysis buffer (100 mM NaCl, 20 mM Tris pH 7.5, 1 mM EDTA, 1 mM EGTA, 1 % Triton X-100, with complete protease inhibitor cocktail (Roche) and phosphatase inihibitor cocktails II and III (Sigma Aldrich)) using a GentleMACS tissue dissociator (Miltenyi) followed by sonication and clarification of homogenates by centrifugation at 10,000 g for 10 minutes. Homogenate protein concentrations were quantified by BCA assay (ThermoFisher Scientific). Cytokine and chemokine levels in homogenates and plasma were quantified by multiplex ELISAs according to manufacturer's instructions (Mesoscale Diagnostics, eBioscience).

RNA was extracted from tumours stored in RNALater using RNeasy mini kits (Qiagen), followed by mRNA reverse transcription with multiScribeTM MuLV enzyme (Life Technologies). Chemokine, cytokine and PD-L1 mRNA expression was quantified using a mouse immune gene signature array (Applied Biosystems #4367786) and a specific PD-L1 Taqman qPCR primer set (Applied Biosystems Mm00452054_m1). qPCR was run on a QuantStudio7 Flex real time PCR system (Applied Biosystems).

### Ex vivo splenocyte re-stimulation

Pan02 cells are MHCI negative (data not shown), and were therefore IFNγ-treated to upregulate MHCI (20 ng/mL, 24h), before mitomycin C treatment (50 μg/mL, 20min) and extensive washing. 8.3 × 10^3^ Pan02 cells were co-cultured with 8.3 × 10^4^ disaggregated splenocytes from Pan02 tumour-bearing mice (day 29 post-implantation) in RPMI (Life technologies) with 10 % fetal bovine serum (SAFC Biosciences) and 10 U/mL IL-2 for 96 hours in Multiscreen_HTS_ IP plates (Millipore) coated with anti-IFNγ capture antibody (eBiosciences). The number of IFNγ-producing T cells was then quantified by ELISPOT assay according to manufacturer's instructions (eBiosciences).

### Statistical analysis

The effect of αCD40 on tumour growth was analysed using a statistical permutation test comparing tumour growth over the whole course of model progression, using the method of [48] with analysis performed using Statmod (https://cran.r-project.org/web/packages/statmod/index.html) in R version 1.4.18. All other statistical analyses were performed in Graphpad Prism v6.03.

## SUPPLEMENTARY FIGURES



## Abbreviations

αCD40: CD40 agonist antibody
PD-1: programmed cell death protein 1
PD-L1: programmed death ligand 1
CTLA-4: cytotoxic lymphocyte-associated antigen 4
T_reg_: regulatory T cell;IFNγ, interferon γ
KPC: KRAS^LSL-G12D/+^ / Trp53^LSL-R172H/+^ / Pdx-1-Cre
i.p.: intraperitoneal
s.c.: subcutaneous
APC: antigen presenting cell
IHC: immunohistochemistry
TME: tumour microenvironment
