# Supplementary material for: Transformation of the tumour microenvironment by a CD40 agonist antibody correlates with improved responses to PD-L1 blockade in a mouse orthotopic pancreatic tumour model
Source: Oncotarget. 2016 Feb 23;7(14):18508–20. doi: 10.18632/oncotarget.7610 (PMC4951305; doi:10.18632/oncotarget.7610)
Supplement: Supplementary file 1 [file oncotarget-07-18508-s001.pdf]

SUPPLEMENTARY FIGURES

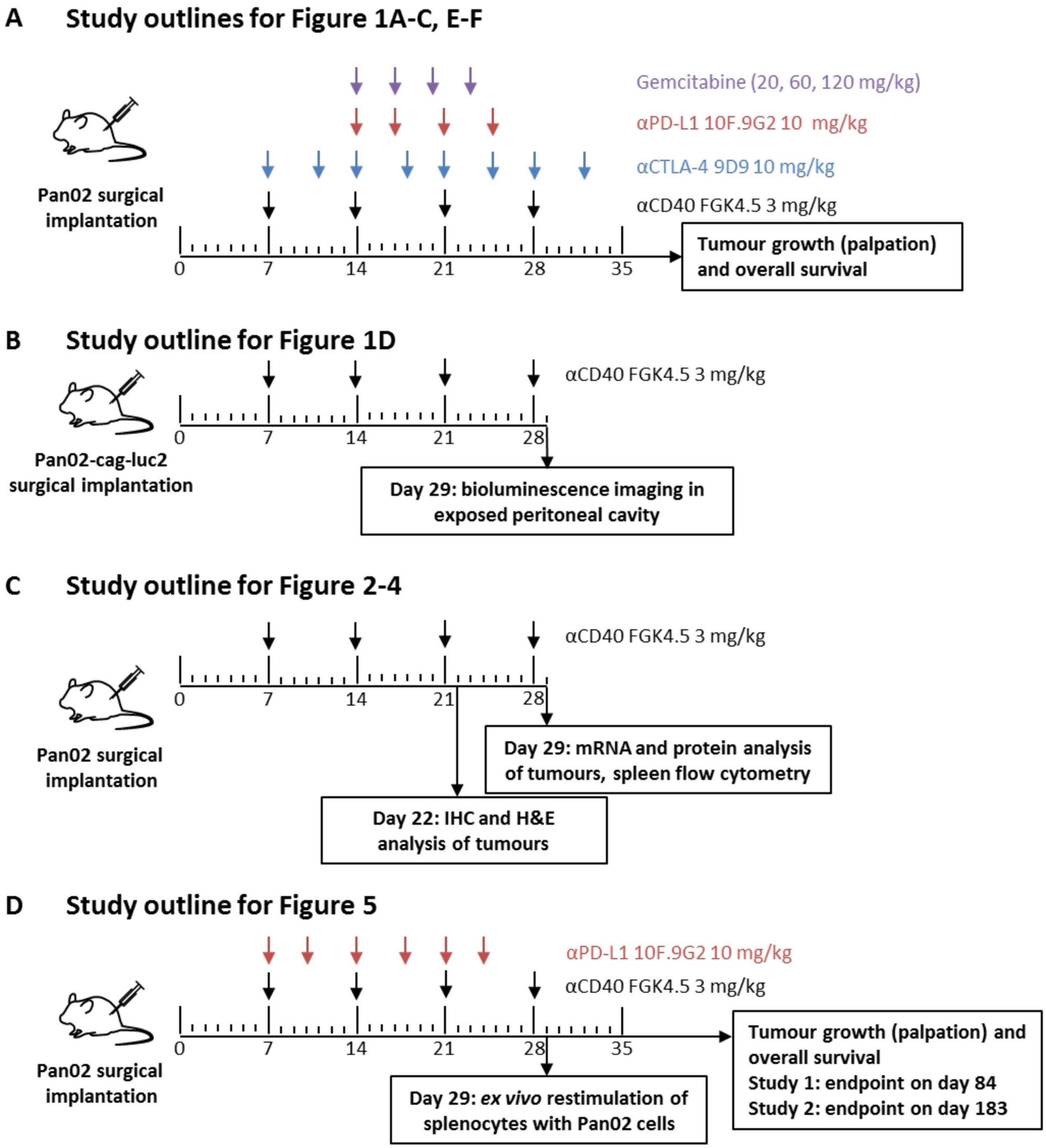

Supplemental Figure S1: Orthotopic Pan02 study outlines A–D. Dosing schedules and endpoints for studies described the main figures. Figure 1A–C, E–F (A) Figure 1D (B) Figures 2–4 (C) Figure 5 (D).

A

Description	Score
Gritty lump, <1mm in diameter	1
>1-2mm diameter	2
>2-5mm diameter	3
>5-10mm diameter	4
>10mm-15 diameter	5
>15mm diameter	6
Secondary indicators associated with disease progression:	
>5% weight loss	+1
Tumour attachment to body wall	+1
Secondary tumours	+1
Abdominal swelling	+1
Eschars/sores (rare)	+1

B

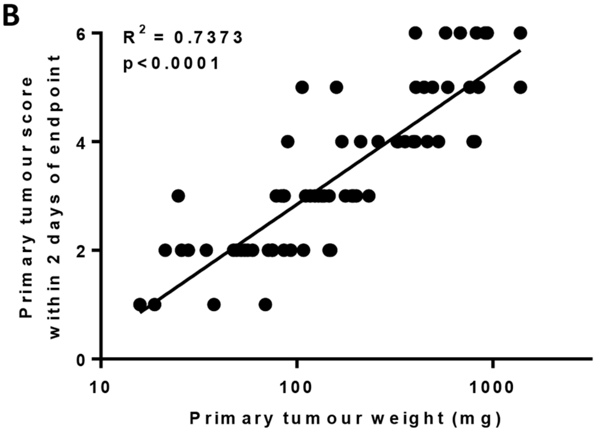

Supplemental Figure S2: Scoring system for orthotopic Pan02 model A. System used for scoring primary orthotopic Pan02 tumour size based on careful abdominal palpation (score of 1 – 6). An additional “secondary” score is assigned based on other indicators associated with disease progression (+1 for each indicator). **B.** Comparison of primary Pan02 tumour score by palpation within two days of endpoint and mass of primary tumour at endpoint. Data are pooled from three independent experiments and from animals treated with isotype control antibody, αCD40, αPD-L1 and αCD40 + αPD-L1 in combination as described in main text, with endpoints at days 22 – 84. n = 76 animals. Linear regression R^2=0.7373, p<0.0001 vs. slope of zero.

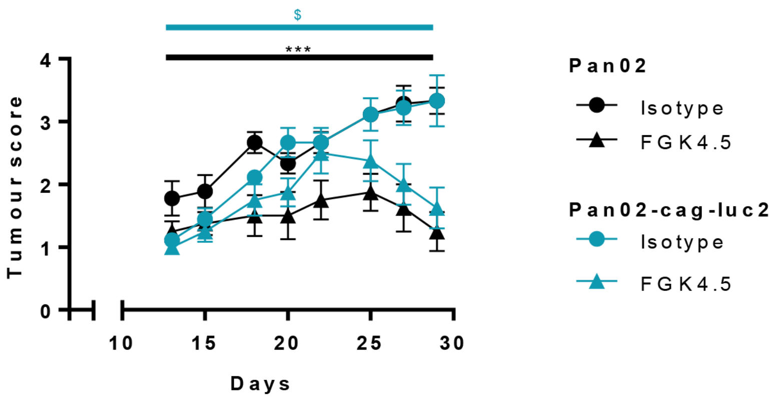

Supplemental Figure S3: αCD40 delays tumour growth in both Pan02 and Pan02-cag-luc2 tumour-bearing mice. Animals bearing Pan02 or Pan02-cag-luc2 tumours were treated with 3 mg/kg FGK4.5 or isotype control rat IgG2a (i.p. once weekly for four weeks), and scored regularly for primary tumour growth as described in Supplemental Figure 2A. Data are mean ± s.e.m. of n ≥ 8 animals per group. ***p=0.001 Pan02 tumour bearing animals treated with isotype control v.s. FGK4.5, $^{\$}$p<0.05 Pan02-cag-luc2 tumour bearing animals treated with isotype control v.s. FGK4.5, statistical permutation test (see materials and methods for details).

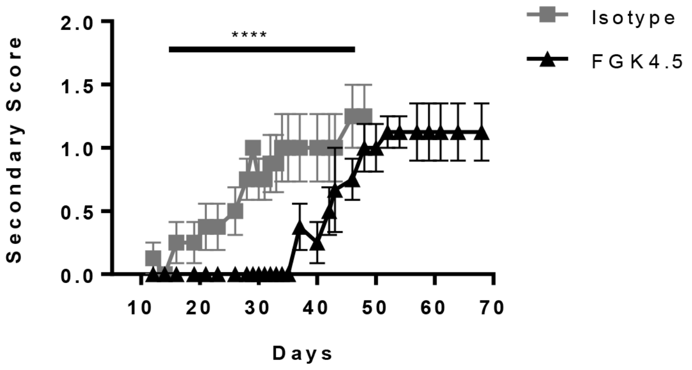

Supplemental Figure S4: αCD40 delays onset of secondary indicators associated with disease progression in Pan02 model. Animals bearing Pan02 tumours were treated with 3 mg/kg FGK4.5 or isotype control rat IgG2a (i.p. once weekly for four weeks), and scored regularly for the presence of secondary indicators associated with disease progression as described in Supplemental Figure 2A. Data are mean ± s.e.m. of n = 8 animals per group. ****$p<0.0001$ statistical permutation test versus isotype control (see materials and methods for details).

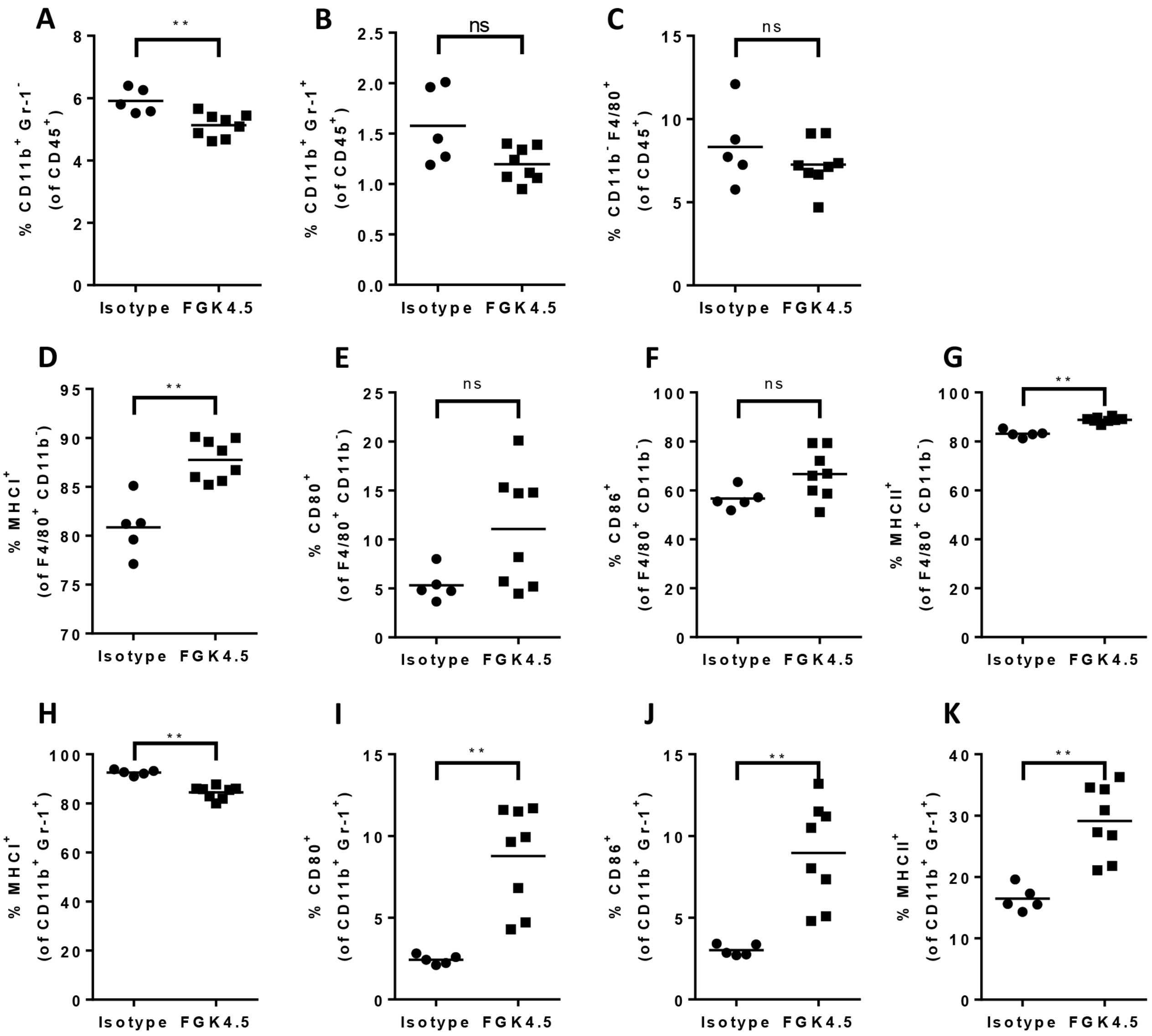

Supplemental Figure S5: αCD40-induced changes in numbers and maturation of splenic myeloid cells A–C. Effect of FGK4.5 (3 mg/kg, day 7, 14, 21, 28, endpoint on day 29) on numbers of $CD11b^{+}$ $Gr\text{-}1^{-}$ (A) $CD11b^{+}$ $Gr\text{-}1^{+}$ (B) and $CD11b^{-}$ $F4/80^{+}$ (C) myeloid cells in spleen. **D–K.** Effect of FGK4.5 on expression of MHCI (D,H) CD80 (E,I), CD86 (F,J) and MHCII (G,K) expression on $F480^{+}$ $CD11b^{-}$ (D-G) and $CD11b^{+}$ $Gr\text{-}1^{+}$ (H-K) myeloid cells. $n \geq 5$ animals / group, $^{**}p<0.01$ Mann-Whitney test.
